# IQOS point-of-sale marketing strategies in Israel: a pilot study

**DOI:** 10.1186/s13584-018-0277-1

**Published:** 2019-01-14

**Authors:** Yael Bar-Zeev, Hagai Levine, Gil Rubinstein, Ihab Khateb, Carla J. Berg

**Affiliations:** 1 Braun School of Public Health and Community Medicine, Hadassah –Hebrew University, Jerusalem, Israel; 2 Centre for Smoking Cessation and Prevention, Division of Community Medicine, Faculty of Health, Ben-Gurion University, Beer-Sheva, Israel; 3 Department of Behavioral Sciences and Heath Education, Rollins School of Public Health, Winship Cancer Institute, Emory University Woodruff Health Sciences Center, 1518 Clifton Road, NE, Room 524, Atlanta, GA 30322 USA

**Keywords:** Tobacco control, Tobacco policy, ENDS, Tobacco marketing

## Abstract

**Background:**

Philip Morris International’s IQOS (“I Quit Ordinary Smoking”) device has increasingly penetrated the global tobacco market. In Israel, among the first countries with IQOS in its market, the IQOS device is sold in specialty stores and online; the heat sticks – HEETS – are sold at traditional retailers. Advertising restrictions in many contexts including Israel have shifted industry marketing efforts to point-of-sale (POS). Given the nuances of IQOS and HEETS product distribution and the importance of POS marketing, we conducted a pilot study of IQOS POS marketing strategies.

**Methods:**

Data collectors assessed product offerings, pricing, promotional strategies, and placement in a sample of 15 IQOS retailers (10 convenience stores, 3 grocery stores, 2 tobacco shops) in three Israeli cities (Beer-Sheva, Haifa, Jerusalem).

**Results:**

All retailers sold cigarettes; many carried other forms of tobacco (e.g., cigar products). Average price for a HEETS package was 30.2 Shekels (SD = 2.7); average price for the least expensive cigarette pack was 27.4 (SD = 1.5). HEETS were on average 9.5% more expensive than cigarettes. Posted ads were uncommon; rather, product displays were prominent. HEETS packages were often placed in a separate display box, at higher and more noticeable positions, and closer to consumers. Additionally, 11 retailers placed cigarettes and 10 placed HEETS near youth-oriented merchandise; 9 retailers placed cigarettes and HEETS, respectively, within 1 m of the floor.

**Conclusions:**

This study represents an initial step in assessing IQOS POS practices – critical in advancing the ability to facilitate related research and regulation of emerging tobacco products in Israel and more broadly.

## Background

The tobacco industry has had a range of products on the market, recently introducing products that heat but do not burn tobacco, referred to as heated tobacco products (HTPs) or “heat-not-burn”. These HTPs include Philip Morris International’s (PMI) “IQOS” (“I Quit Ordinary Smoking”), British American Tobacco’s “glo”, and Japan Tobacco International’s “TECH” [1]. Moreover, recent research has demonstrated interest in HTPs have eclipsed interest in other electronic nicotine delivery systems (ENDS), as reflected by online media search activity, suggesting the global expansion of HTPs and related interest [2].

The product that has the greatest market share of HTPs, as well as research on its use and impact, is IQOS [3, 4]. The release of PMI’s IQOS product began in Japan, Italy, and Switzerland; now a total of 30 some countries have them in their markets [1, 5]. Many countries will face regulatory implications related to new tobacco products such as HTPs. Public health policies must be informed by an understanding of the tobacco industry’s target markets and marketing strategies aimed at shaping perceptions of their products and their use. These marketing strategies are informed by multiple types of market research and are a reflection of how and where the tobacco industry displays their products, what messaging and other strategies are used to promote their products, and pricing strategies, among other strategies [6, 7]. Particularly relevant, the US Food and Drug Administration (FDA) pre-market review of new tobacco products, such as HTPs, requires examples of product package labels and ads, along with data showing their likely influence on consumer behaviors.

IQOS was released in Israel on December 2016, offering an opportunity to learn about PMI’s marketing strategies for IQOS and advance readiness to regulate IQOS in other countries [5, 8]. In Israel, two major campaigns have been identified – one focused on policy makers and one on the public – with major campaign elements focusing on IQOS being a fundamentally different product from traditional cigarettes with the potential for harm reduction, thus warranting different regulation and policies (e.g., taxation) [5]. Indeed, this is a pivotal time for understanding all major aspects of IQOS marketing.

IQOS device distribution in Israel is unique in that IQOS devices and accessories (e.g., cleaning sticks, leather wrap) can generally be purchased in only two ways – by purchasing them at IQOS specialty stores or by ordering the device online, which then results in in-person delivery and education about the device. The heat sticks – HEETS – are then purchased at traditional retailers (e.g., convenience stores, gas stations) [9]. Thus, the point-of-sale (POS) environment is a critical part of understanding how consumers are introduced to IQOS and HEETS, and indeed, consumers may encounter HEETS before IQOS given this market structure.

We conducted a pilot study of IQOS POS marketing strategies, adapting the Standardized Tobacco Assessment for Retail Settings (STARS) [10] and assessing a subset of IQOS retailers in three metropolitan areas in Israel.

## Methods

### Study setting and sample selection

Israel was chosen as the site of this study because of its established IQOS market, with approximately 3250 IQOS retailers nationally [9]. Additionally, Israel is a democratic, multi-ethnic, high-income country [11] with a parliamentary, multiparty system and a modern market-based economy, suggesting that data derived here may be relevant to other countries likely to face IQOS market penetration. Regarding tobacco control, Israel signed and ratified the World Health Organization Framework Convention on Tobacco Control (FCTC) in 2003 and 2005, respectively. Currently, there are bans on billboard, radio, and TV advertising; advertising is allowed in certain sections of the press and on the internet [12]. At POS, there are no restrictions on advertising, but signage indicating minimum age for purchase (18 years) is required. In April 2017, after an appeal to the Israeli Supreme Court, IQOS was declared as a tobacco product, resulting in its being subject to the same marketing restrictions as other cigarettes. Given the restrictions on marketing in the regular media POS marketing is a critical marketing opportunity for IQOS. In January 2018, after an additional appeal, IQOS was taxed at the rate of other cigarettes [8].

In Spring 2018, two trained data collectors drew a sample of up to 7 retailers from three cities with high volumes of IQOS retailers (Beer-Sheva with 158 IQOS retailers; Haifa with 367 retailers; and Jerusalem with 310 retailers), using data from the PMI website [9] until the target sample size for this pilot (*N* = 15) was reached.

### IQOS POS assessment tool

Based on the Standardized Tobacco Assessment for Retail Settings (STARS), which demonstrates high reliability [10], a similar assessment tool was developed to include HTPs, specifically IQOS, as IQOS is presently the only HTP in the Israeli market. The three authors developed the tool to include measures based on content relevance and reliability, adapted to include HTPs.

The final assessment tool assessed: 1) ***store characteristics*** (i.e., type of store, signage indicating minimum age requirements for purchasing tobacco); 2) ***products*** sold (i.e., cigarettes, little cigars or cigarillos [LCCs], large cigars, smokeless tobacco [e.g., chew, dip, snus], electronic nicotine delivery systems [ENDS] other than HTPs, HTPs which were exclusively IQOS; 3) ***price*** (i.e., least expensive price of a cigarette pack and HEETS) and any price promotions across products; 4) ***promotional strategies***, including product advertisements outside or inside of the store, ability to sample products, and special promotions for populations (e.g., military, college students) or loyalty club memberships; and 5) ***placement***, including IQOS displays, placement near to youth-oriented merchandise (specifically placement of products within 30 cm of toys, candy, gum, slushy/soda machines, or ice cream), and whether products were displayed within one meter of the floor (which aligns with the field of vision of youth).

### Data collection

The data collectors were trained to use the assessment tool by the lead author (YBZ). Free text notes were filled in with comments and information provided by the merchants. In particular, data collectors noted merchant comments regarding IQOS product safety/harm, potential cessation or harm reduction benefits, and benefits related to secondhand smoke/smell.

### Data analysis

Given the study’s exploratory nature and small sample size, we used SPSS 23.0 to conduct descriptive statistics only.

## Results

### Store characteristics

Table 1 present data from the POS assessments across the 15 IQOS retailers. The Beer-Sheva sample consisted of 5 convenience stores. The Haifa sample consisted of 3 convenience stores, 3 grocery stores, and 1 tobacco shop. The Jerusalem sample consisted of 2 convenience stores and a tobacco shop.

**Table 1:** IQOS POS Characteristics

Variable	N (%) or M (SD)
City, N (%)	
Beer-Sheva	5 (33.3)
Haifa	7 (46.7)
Jerusalem	3 (20.0)
Type of store, N (%)	
Convenience store at a gas station	5 (33.3)
Convenience store not at a gas station	5 (33.3)
Grocery store	3 (20.0)
Tobacco shop	2 (13.3)
Minimum age signage, N (%)	
Minimum age to purchase tobacco signage	10 (66.7)
Minimum age to purchase IQOS signage	6 (40.0)
Least expensive price (ave.) in Shekels, M (SD)	
Cigarette pack	30.2 (2.7)
HEETS	27.4 (1.5)
Placement, N (%)	
Placed near youth-oriented merchandise	
Cigarettes	11 (73.3)
HEETS	10 (66.7)
Placed within 1 m from floor	
Cigarettes	9 (60.0)
HEETS	9 (60.0)

Ten (66.7%) of retailers posted signage indicating the minimum age to purchase tobacco products; 6 (40.0%) of retailers posted minimum age signage specific to purchasing HTPs.

### Products

All retailers sold cigarettes, 10 LCCs, 9 large cigars, 10 smokeless tobacco, 2 ENDS, and 11 nargila/waterpipe/hookah.

### Pricing and price promotions

The average price for the least expensive HEETS purchase was 30.2 Shekels (SD = 2.7); whereas, the average price for the least expensive cigarette pack was 27.4 (SD = 1.5). We also examined the price of HEETS relative to the least expensive price for a PMI cigarette pack (most commonly “Next”). In all but one retailer, HEETS were sold at a higher price, on average 9.5% more expensively, with the price difference ranging from 1 to 11 Shekels. Price promotions were rare in general (2 retailers offered them for cigarettes; none offered for other products).

### Other promotional strategies

Exterior advertisements were uncommon, with only 3 having cigarette ads, 1 for smokeless tobacco, 1 for ENDS, and 2 for nargila/waterpipe/hookah. Interior ads were also uncommon; rather, product displays were prominent. No retailer had specific ads for HEETS. Free samples were not provided, and no retailers offered any special promotions for populations (e.g., military, college students) or loyalty club memberships.

### Placement

Eleven retailers placed cigarettes and 10 placed HTPs near youth-oriented merchandise. Also, 9 retailers placed cigarettes and HTPs, respectively, within 1 m of the floor. Further, in 8 retailers, HEETS packages were separated from other tobacco products sold, and placed in a separate display box. In 2 retailers, HEETS displays were placed at higher and more prominent positions, closer to consumers, and with color coding to differentiate the tobacco flavoring and strength.

### Merchant attitudes

When asked directly, retailers stated HTPs were less harmful (26.7%), can be used as a method for quitting or reducing number of conventional cigarettes smoked (13.3%), and do not emit smoke and produce less adverse smell (40.0%).

## Discussion

This study was a first step in adapting a surveillance tool for assessing the marketing practices of IQOS retailers in a country, specifically in Israel. It provided several important findings that should be integrated into the assessment of the overall marketing strategy of IQOS in Israel [5]. First, most retailers adhered to regulations; for example, two-thirds posted signage regarding minimum age to purchase tobacco; however, only two-fifths had this signage specified for HTPs. Moreover, we did not observe free samples.

Second, pricing strategies were noteworthy. We found that HEETS were not priced competitively in relation to cigarette packs in general and were priced an average 9.5% more expensive, with the price difference ranging from 1 to 11 Shekels. Price elasticity is a critical consideration related to product choice [13]; thus, such pricing differentials may disincentivize individuals to try or switch to IQOS, which may be further compounded by the inconvenience related to acquiring the IQOS device itself.

Third, findings indicated that HEETS were often displayed prominently and often in places likely to be viewed by youth. Regulatory efforts are needed to ensure that HEETS and other tobacco products are not easily viewed or targeted toward youth [14].

The limitations of this study include its focus on a convenience sample of 15 retailers chosen from the PMI IQOS website in three Israeli cities, the use of a single auditor, and a limited scope in assessment. Subsequent research will test inter-rater reliability, examine the utility of the instrument across multiple settings, and identify specific variables relevant to other settings/countries with different regulations regarding tobacco and HTPs specifically.

## Conclusions

This study represents an initial step in developing an assessment tool for understanding marketing and POS practices for HTPs, specifically IQOS. Future research will validate the tool across contexts and examine its reliability. Tools such as this are needed to facilitate research regarding the impact of POS marketing on use of IQOS and other emerging tobacco products and to inform our understanding of the broader IQOS marketing strategies across countries.
